# Can resistance prehabilitation training bring additional benefits in valvular cardiac surgery? protocol for a randomized controlled trial

**DOI:** 10.1371/journal.pone.0303163

**Published:** 2024-05-07

**Authors:** Jorge Montero-Cámara, Francisco José Ferrer-Sargues, María José Segrera Rovira, Adrián Sarria Cabello, David Cuesta Peredo, Juan Antonio Margarit Calabuig, Noemí Valtueña-Gimeno, María Luz Sánchez-Sánchez

**Affiliations:** 1 Deparment of Nursing and Physiotherapy, Universidad Cardenal Herrera-CEU, CEU Universities, Alfara del Patriarca, Valencia, Spain; 2 Hospital Universitario de la Ribera, Alzira, Valencia, Spain; 3 Department of Physiotherapy, Physiotherapy in Motion, Multispeciality Research Group (PTinMOTION), University of Valencia, Valencia, Spain; Stanford University School of Medicine, UNITED STATES

## Abstract

**Introduction:**

Cardiovascular diseases (CVD) are a group of illnesses that include coronary heart disease, cerebrovascular disease, congenital heart disease and deep vein thrombosis. Major surgery is often chosen as the treatment of choice for CVD. The concept of fast-track rehabilitation after surgery appeared in the 1970s. Participation in these exercise-based prehabilitation programmes may decrease postoperative complications and length of hospital stay. The primary aim of the present study is to evaluate whether the implementation of an additional resistance training (RT) prehabilitation protocol within cardiac exercises based prehabilitation can reduce intensive care unit (ICU) length of stay, postoperative complications and hospital length of stay (LOS).

**Methods:**

A protocol of a prospective, parallel, randomised clinical trial includes 96 adult patients diagnosed with valvular pathology and who have been scheduled for surgery. The participants will be randomly assigned to two groups of 48. Control group will be treated with ventilatory and strengthening of respiratory muscles, and aerobic exercise. Experimental group, in addition, will be treated with RT of peripheral muscles. Both hospital stay and ICU stay will be assessed as main variables. Other secondary variables such as exercise capacity, quality of life and respiratory values will also be assessed. Quantitative variables will be analysed with a T-Test or ANOVA, or Mann Witney if the distribution is non-parametric.

**Results and conclusion:**

This will be the first controlled clinical study focused on adding strength exercise as an additional treatment during prehabilitation. The results of this study will focus on helping to improve rehabilitation and prehabilitation protocols, considering that it is essential to maintain pulmonary training, as well as the inclusion of peripheral exercises that help people with heart disease to be in a better physical condition in order to increase their participation and sense of quality of life.

## Introduction

Cardiovascular diseases (CVD) are a group of illnesses that include coronary heart disease, cerebrovascular disease, congenital heart disease, deep vein thrombosis, pulmonary embolism, and disorders of the heart and blood vessels [1]. A number of risk factors are commonly associated with acute CVDs [1,2] and, in terms of socio-demographic level, poorer economic or educational conditions may contribute to increased risk factors [3]. CVD are the leading cause of mortality worldwide, representing 31% of deaths, most of them because of coronary heart disease (specifically 19% in men and 20% in women) [1,4]. In Spain, CVD caused 24% of all deaths in 2020 [5].

Major surgery is often chosen as the treatment of choice for CVD, with patients often classified as "high-risk" due to comorbidities such as frailty, obesity or advanced age [6]. These patient characteristics may increase the likelihood of different postoperative pulmonary complications [7,8].

The concept of *fast-track* rehabilitation after surgery (ERAS, Enhanced Recovery After Surgery) combines different measures to optimise the patient’s condition before, during and after surgery, to allow them to recover their maximum functional capacities as quickly as possible [9–12]. The ERAS protocol represents a model of patient-centred healthcare collaboration, with preoperative preparation and postoperative re-education [9,11,13]. Furthermore, participation in these exercise-based prehabilitation programmes (EBPrehab) may decrease postoperative complications and length of hospital stay (LOS) [9] improving patients’ functional capacity and decreasing healthcare costs [14]. The most commonly used exercises in the pre-surgical intervention include aerobic training [10,12,15], chest expansion exercises, ventilatory work with devices [9], and exercises to strengthen the respiratory muscles [16,17]. This combined work of the exercises can reduce complications and LOS [12].

Postoperative acquired weakness syndrome significantly affects cardiovascular patients following surgery and their ICU stay [18]. This condition has a pronounced impact on peripheral musculature. Research indicates that lower preoperative muscle density is associated with increased postoperative mortality rates [19–21] and a higher likelihood of pulmonary complications [20,22–24]. Additionally, reduced manual grip strength correlates with higher mortality, longer hospital stays, and increased risk of postoperative complications [21,23,24].

An exercise programme that includes peripheral resistance training (RT) has been shown to be effective in reducing in-hospital mortality and LOS in surgical patients [25,26]. Regarding cardiovascular disease, RT added to aerobic training can improve quality of life and left ventricular function in people with coronary disease [27] and chronic heart failure [28]. However, despite the wide variety of cardiac prehabilitation programmes, to our knowledge none of those published have included RT in a specific subgroup of cardiovascular disease, such as valve surgery. Therefore, the purpose of the present study is to evaluate whether the implementation of an additional RT prehabilitation protocol within a cardiac EBPrehab (based on aerobic training, ventilatory work and respiratory muscle strengthening) can reduce ICU LOS. In addition, postoperative complications and hospital LOS in valvular patients undergoing cardiac surgery will be measured. Finally, a secondary objective is to determine whether a programme that includes RT in addition to respiratory and aerobic training can have better effects on ventilatory variables than a traditional programme based on aerobic exercise, ventilatory work, and respiratory muscle strengthening.

## Methods

### Study design and ethical approval

A prospective, parallel, randomised, single-centre, clinical trial protocol has been designed. This study complies with the ethical principles established in the Declaration of Helsinki and was approved by the Ethics Committee of the Hospital de La Ribera (code PI 04-05/23). The protocol was registered in the U.S. National Library of Medicine (ClinicalTrials.gov) with identifier NTC05911191. The Hospital de La Ribera is subject to public funding, and this protocol does not require specific funding, as it does not entail any additional cost for the Hospital de La Ribera or for the researchers. The authors did not receive specific funding for this work. The funders did not and will not have a role in study design, data collection and analysis, decision to publish, or preparation of the manuscript.

All candidates will be given and explained an information sheet, which will accompany the informed consent document, with a detailed explanation of the study. After reading and explanation, they will be asked to sign if they consent to participate. All individuals in this manuscript have given written informed consent (as outlined in PLOS consent form) to publish these case details.

### Participants: Eligibility criteria and recruitment

Ninety-six patients will participate in this study and their recruitment is expected to continue from September 2023 until July 2025. Inclusion criteria for the sample will be adult patients diagnosed with valvular pathology who have been admitted as candidates for cardiac surgery for the first time at the Hospital de La Ribera, and who have been scheduled for surgery. Only those who voluntarily agree to participate in the study and sign the informed consent form will be admitted. Patients with the following characteristics will be excluded: (a) stage 4 or 5 renal failure, (b) low ejection fraction, (c) Euroscore greater than 15 [29], (d) non-ST-segment elevation acute coronary syndrome (NSTEACS), (e) coronary artery disease, (f) or need for urgent intervention. Those with cognitive deficit, diagnosis of pulmonary disease, heart failure, unstable angina, arrhythmias, severe aortic stenosis, mitral regurgitation, aortic aneurysm, recent embolism, recent myocarditis or pericarditis, or comorbidities limiting the performance of the functional test will also be excluded.

All patients referred to the Physiotherapy Unit from the Cardiac Surgery Unit of the Hospital de La Ribera (Alzira, Spain) will be considered candidates for this study, provided they attend the initial physiotherapy assessment. Recruitment will, therefore, be carried out among these attendees, and eligibility will be confirmed by a physiotherapist. Signed informed consent will be obtained prior to any measurement or intervention. Participants will be randomly assigned to either the experimental prehabilitation group or the conventional prehabilitation group. The random allocation of participants will be done using the Epidat 4.2. software, ensuring a size of 48 participants in each of the experimental and control groups [30]. Each participant will receive a correlative numerical code according to their order of arrival at the first appointment. These numerical codes will be randomly assigned to one or the other group before the start of the study. A computer generated random-number table will be used for allocation.

The assessments will be conducted by three physiotherapists who will be intentionally kept unaware of the group assignments for each participant. Participants will be explicitly instructed not to reveal any details about the specific exercises they undertook during their pre-surgery treatment. Additionally, the physiotherapist overseeing the groups will not be responsible for conducting the measurements. Prior to measurements, the physiotherapists will undergo a training session to standardize measurement criteria for each variable. The allocation of participants will remain concealed from both patients and the physiotherapists responsible for conducting measurements during the patient’s hospital stay. Only the principal investigator of the study will have knowledge of the allocation, and patients will be instructed not to reveal details about the type of training prior to surgery.

### Intervention

The timeline for the development of the intervention can be seen in the first figure (S1 Fig), while the second figure (S2 Fig) shows a flow chart of how the intervention and measurements will be developed in each group. Both groups will receive a first session in which they will be instructed to carry out the unsupervised home EBPrehab programme, consisting of respiratory training, ventilatory training, strengthening of the respiratory muscles, aerobic endurance exercise through continuous walking, and a series of recommendations on post-surgical care. In addition, explanatory videos with all the information about the home exercises will be provided so that they can consult them whenever they need it. Furthermore, a preoperative musculoskeletal and cardiopulmonary rehabilitation programme focused on RT will be implemented in the experimental group. The intervention has been designed following the American College of Sports Medicine (ACSM) guidelines for exercise prescription, considering the FITT-VP principles (Frequency, Intensity, Type, Time, Volume, Progression) for patients with cardiorespiratory pathology and adjusting them to the surgical population [31]. This peripheral muscle strengthening program (Table 1) will be modified and adapted in each classroom session, which will require a weekly evaluation and modifications to the load and the number of series and/or repetitions, depending on each participant. Participants will also be educated to adjust the intensity using the Borg CR-10 scale [32], asking them to maintain a moderate intensity, between four and seven on this scale.

**Table 1:** Physiotherapy intervention in the intervention and control groups. Own source.

**Common interventions for both groups**	
A single explanatory session of: Ventilatory breathing exercises. • Diaphragmatic breathing. • Thoracic expansion exercises. • Incentive spirometer. • Expiratory flow technique. Exercises to strengthen the respiratory muscles. Post-surgical care. Pre-surgical cardiopulmonary rehabilitation exercises (continuous walking). Use of the Borg CR-10 scale.	
**Control group**	**Experimental group**
Application at home of what was learnt in the initial explanatory session. Unsupervised follow-up. Registration of the activity with Google Fit.	Strength training of peripheral muscles. Application at home of what was learnt in the initial explanatory session. Supervised monitoring and weekly adjustment of resistance. Recording of home activity with Google Fit.

The prehabilitation programme will be structured into three different phases (Table 2). Each participant will receive a list of links to the demonstration videos and instructions about the performance of each exercise. All the videos can be viewed by clicking on the following link: https://www.youtube.com/playlist?list=PLDt-M0gErNrx2Bp2x4FPSRSiOUqJUL—y. The individuals in this manuscript have given written informed consent (as outlined in PLOS consent form) to publish these case details.

**Table 2:** Physiotherapy intervention.

**Training common to both groups**					**Specific training of the experimental group**			
**Respiratory-training phase**				**Endurance-training phase**	**Peripheral resistance-training phase**			
20 minutes				1 minute, increasing 5 minutes in each training session, until 20 minutes.	10 minutes, increasing 5 minutes in each training session, until 20 minutes.			
**Exercise**	**Series**	**Reps.**	**Rest**	**Exercise**	**Exercise**	**Series**	**Reps.**	**Rest**
Diaphragmatic breathing	3	10	1 min.	Walk in continue modality. Perceived exertion will be important, so we will give a modified-Borg scale to all patients.	Sit-to-stand	3	8–12	20 sec
Thoracic expansion	3	10	1 min.		Shoulder bridge	3	8–12	20 sec
Incentive spirometry	1	10 25% MC	1 min.		Shoulder press	3	8–12	20 sec
Incentive spirometry	1	1 100% MC	1 min.		Biceps curl	3	8–12	20 sec
Expiratory flow techniques			1 min.					
**Respiratory strengthening**								
Inspiratory muscle training	1	30% MIP	1 min.					

Abbreviations: Repetitions (Reps), maximum capacity (MC), maximum inspiratory pressure (MIP), minute (min), second (sec).

1. Breathing training phase (both groups, twenty-one minutes). The breathing intervention will include diaphragmatic breathing (three sets of ten repetitions), chest expansion exercises (three sets of ten repetitions), and incentive spirometry with the Portex® Coach 2® Incentive Spirometer 4000 ml (one set, with ten repetitions up to 25% of maximum capacity, and only one repetition at maximum inspiratory capacity). Expiratory flow techniques will be taught, using the Sibelmed® Datospir Peak-10 incentive device.
2. Aerobic endurance training phase (both groups, ten minutes, increasing by five minutes at each home training session, up to twenty minutes). Each patient will walk in an harmonic continuous modality, following the minimum recommendations of the World Health Organisation [33]. Perception of exertion will be assessed using a modified Borg scale. The aerobic endurance training should be practised every day until the surgery.
3. Strengthening phase
  1. Respiratory strengthening (both groups). Specific training of the respiratory muscles will be carried out using the Threshold® Inspiratory Muscle Trainer (IMT) (Respironics, NJ 07054, USA), adjusting the workload to a minimum of 30% of the subject’s maximum static inspiratory pressure. [16,34]. The workload of the IMT device ranges from 9 to 41 cmH_2_O. The protocol will contain twenty-one minutes of training divided into seven sets, with two minutes of work and one minute of rest between the sets. Each participant will receive a list of links to the demonstration videos and instructions about the performance of each exercise.
  2. RT phase (only in the experimental group, ten minutes, increasing by five minutes in each training session, up to twenty minutes). In each hospital session, participants will complete three sets of four exercises. During the first sessions, training will consist of eight repetitions of the exercises, performed with light resistance bands, and emphasising analytical training of the main muscles (deltoids, biceps brachii, triceps brachii, abdominals, trunk extensors, quadriceps, hamstrings and calves) [35]. After the second training session in the hospital, progress to using medium-resistance bands and/or increasing repetitions for each exercise will be used. In the final phase, the volume will increase to twelve repetitions. Each exercise will have a rest period of 60 seconds.

To verify that participants perform the exercises at home, regardless of the group to which they are assigned, they will be asked to record their activities at home using the Google Fit application [36]. In addition, to encourage adherence, a researcher will contact them weekly by telephone to record whether they have completed their exercises and if they have had any difficulties [37].

The prehabilitation programme common to all participants is the same programme that is conventionally carried out in the prehabilitation service of Hospital La Ribera. Regarding the RT, although there is no evidence that the exercises proposed before surgery can cause harm to cardiac patients awaiting surgery, to minimise the risks, the first session will be carried out at the hospital, adapting the intensity to each patient every week before surgery. For the adaptations of the resistance exercises to be carried out by the experimental group, it will be necessary for the participants to visit the hospital at least once a week. Any adverse events perceived by the patient during the home sessions will be reported. All adverse events will be evaluated, recorded, and discussed in the final manuscript.

### Measurement procedures and outcomes

The variables will be divided into four different areas, including LOS, quality of life, respiratory values, and exercise capacity (Table 3).

**Table 3:** Outcome tools for measures and time.

	Hospital stay	Quality of life	Respiratory values	Exercise capacity
**Outcomes**	Hospital stay since surgery to discharge: Critical care Unity (ICU). Hospital stay out of ICU. Total hospital stay.	Quality of life prior to surgery and six months after surgery	Peak expiratory flow (*1). Inspiratory capacity (*1). Maximum static inspiratory (MIP) (*2). Maximum static expiratory (MEP) (*2).	Aerobic resistance (*2). Lower limb strength (*2). Manual grip strength (*1,2). Biceps brachii and cuadriceps stregth capacity (*1,2).
**Tool**	Accounting of the days according to the data reflected in the medical history.	EuroQoL-5D questionnaire	Sibelmed Datospir Peak-10. Portex Coach 2 Incentive Spirometer 4000ml. MicroRPM device (Carefusion)	2 minute step test Short Physical Performance Battery. Activity logging via Google Fit. Hand dynamometry. Dinamometer Lafayette
**Time**	At hospital discharge	Pre-surgery and 6 months after discharge.	*1.- First appointment 24h after the surgery, at the hospital discharge. *2.- First appointment and a month after the surgery.	

Abbreviations: Intensive care unit (ICU), maximum inspiratory pressure (MIP), maximum expiratory pressure (MEP).

1. LOS. LOS will be measured in hours and minutes, so that stays of less than 24 hours can be better accounted for. The following three variables will be recorded:
  - ICU length of stay will be counted from the end of the surgical procedure until transfer to the patient’s hospital room.
  - Hospital room length of stay will be counted until discharge from the hospital.
  - Complete hospital length of stay will be counted from the end of the surgical intervention until discharge from the hospital.
2. Quality of life. Perception of quality of life will be recorded and assessed both before and in a follow-up, six months after surgery. The EuroQOL-5D quality of life questionnaire, which is valid and reliable for analysing the quality of life in this type of patient [38], will be used. The participant assesses their capacity for self-care, mobility, activities of daily living, the presence of pain or anxiety/depression in categorical levels of severity (no problems, some problems or moderate problems and severe problems) and their health perception using a visual analogue scale [38]. As there are no published data on the inter- examiner reliability of this tool [39], a single nurse will be responsible for collecting these values.
3. Respiratory values. Respiratory values will be collected in three different times: before surgery, 24h after surgery, and on the day of hospital discharge. These tests shall be repeated until three acceptable measurements are obtained (difference <10%), with a one-minute rest between them, and the highest value shall be recorded [40,41]. The three measurements shall be made of:
  - Inspiratory capacity (defined as the volume of air that can be inspired by the resting inspiratory position). With the patient in a seated position, the Portex® Coach 2® 4000 ml incentive spirometer will be used to measure inspiratory capacity, and the value of the variable will be expressed in millilitres. The patient will be instructed to take a deep, unforced inspiration, trying not to exceed the flow mark [42].
  - Peak expiratory flow: This is defined as the maximum flow that a person can exhale during a brief maximal expiratory effort after full inspiration. With the patient in a seated position, the Sibelmed® Datospir Peak-10 device will be used to record the peak expiratory flow, and the value of the variable will be expressed in litres per second. The patient will be instructed in a maximal forced expiration, preceded by a deep nasal inspiration up to inspiratory reserve volume [43].
4. Exercise capacity. Exercise capacity and endurance will be collected before surgery, that is, at the time of the first appointment with Physiotherapy, and one month after surgery. The tests used will be:
  - Aerobic capacity: will be assessed by the 2-minutes Step Test, a valid tool to assess functionality in patients with cardiac pathology after surgery and sensitive to assess perioperative changes, which also has excellent inter-examiner reliability [44]. Following the protocol established by Jones and Rikli [45], implemented in 2022 by Chow et al. in revascularised individuals [44], it evaluates the number of repetitions of knee lifting (between patella and iliac crest) that each participant is able to perform in two minutes. If a person requires technical assistance to perform the test, a stable chair will be placed close to him/her to limit the risk of falling. The assessor shall use a manual counter to record the number of steps each person takes by lifting one knee to the middle distance between the patella and the anterior superior iliac spine [45].
5. Strength capacity.
  - Peripheral strengthening. Measurements to quantify strength shall be taken in both groups at the first physiotherapy appointment and one month after surgery. Likewise, dynamometric measurements shall also be taken 24 hours after surgery.
    - Manual grip strength: grip strength capacity shall be recorded with manual dynamometry (kg), using a Jamar Plus+® device, which is reliable for measuring changes in patients with cardiac disease, and whose minimum detectable change is 5.2 kg and 5.1 kg for the right and left hand, respectively [46]. With the patient in a seated position, he or she will be asked for a maximum hand closure for approximately 2–3 seconds, change hands, and repeat the operation up to three times, with a difference of less than 10% between measurements. This tool has inter- examiner reliability [47].
    - Analytical strength of the biceps brachii and quadriceps femoris: The strength of the biceps brachii and quadriceps femoris will be measured on the arms and legs with a Dynamometer Lafayette Manual Muscle Tester (Lafayette, IN, USA) [48,49]. The measurement technique was described by Bohannon, who verified the excellent intra- and inter-examiner reliability of this tool [50].
    - Lower extremity strength: lower extremity strength will be assessed using the Short Physical Performance Battery, a simple, valid, reliable functional test battery, sensitive to clinical functional changes and with adequate intra- and inter-examiner reliability [51,52]. The test has three parts:
      1. A five-squat test in a standing position, reliable for measuring change, with a minimum detectable change of 3.12 seconds [46]. The time taken to perform the five squats will be categorised according to the following criteria: time less than or equal to 11.19 seconds; between 11.20 and 13.69 seconds; between 13.70 and 16.69 seconds; between 16.70 and 60 seconds; greater than 60 seconds or inability to perform.
      2. Static balance, where the participant is asked to adopt three positions while standing: feet together, semi-tandem and, tandem. It is quantified if the time they can hold the position is less than or at least 10 seconds.
      3. Gait ability, where the patient will be asked to walk in a 4 metres corridor at a normal pace and, the best time (in seconds) of two attempts will be recorded. Gait ability will be categorised as follows: Unable to perform, time greater than 8.7 seconds; time less than 8.7 seconds, but equal to or greater than 6.21 seconds; time less than 6.20 seconds, but greater than or equal to 4.82 seconds; time less than 4.82 seconds.
  - Strength of respiratory musculature.
    - Respiratory pressures: These are used to calculate the strength of the respiratory muscles, both at diaphragmatic (inspiratory) and abdominal and intercostal (expiratory) levels. Maximum static inspiratory and expiratory pressure will be measured in the sitting position, using a MicroRPM device (Carefusion, VYAIRE MEDICAL, UK). Careful explanation of the test will be provided, encouraging the patient to perform a maximum inspiration and inspiration of approximately 2–3 seconds against the resistive device. Predicted values will be estimated using the equation proposed by Heinzmann et al. [53].

The process of intervention and measurement of the variables has been summarised in an explanatory diagram (S3 Fig).

## Statistical analysis

The statistical analysis will be conducted by an independent statistician who will be operating under a strict blinding protocol. This ensures that the statistician remains completely unaware of the group assignments of the participants and the specific treatments administered to each group. All necessary data will be extracted into an Excel spreadsheet and analysed using IBM® SPSS® Statistics 27.0.1.0.

The Kolmogorov-Smirnov test will be used to check whether the data fit a normal distribution. Descriptive analysis will be conducted to explore the variables related to the study.

Quantitative variables with parametric distribution will be assessed with a T-test or ANOVA, whereas if the distribution is non-parametric, the appropriate non-parametric test will be used. This analysis will examine whether there are differences between the groups that may support or refute the hypothesis that strength training reduces ICU stay, hospital stay, and/or complications in this type of patient. This analysis will also be used to test whether ventilation and respiration values as well as quality of life values are different in each group.

To assess whether there is an influence of confounding variables (BMI, toxic habits, comorbidities, underlying pathologies) on the direction of the dependent variables, multiple regression will be performed.

### Sample size

Sample size estimation was performed using G*Power v. 3.1.9.4. software. For the primary outcome (time in intensive care unit), an effect size of 0.613 was determined, based on previous research on the effect of multidimensional preoperative intervention in patients awaiting elective coronary artery bypass graft surgery [50]. To obtain a 0.8 statistical power with a 2-tailed alpha of 0.05 in a Student’s t-test, considering a drop-out rate of 10%, a total of 96 participants will be needed (48 subjects per group).

### Data collection

Sex, age, underlying pathology, comorbidities, body mass index (BMI), and toxic habits will be the descriptive variables collected from both groups. All these data will be stored in their clinical history and will be extracted by the person in charge of epidemiological data management at the hospital. The epidemiologist will include them in an Excel table, also indicating whether each participant survives or not, as well as the information related to the intervention and pathology, catalogued using the International Classification of Diseases 9 (ICD-9) or the appropriate one if there are updates. Days of hospitalisation, intensive care unit (ICU) stay, if applicable, Charlson comorbidity index, and possible postoperative complications for each participant will also be reflected in this table.

Although the database from which the data will be extracted will not contain personal data, as each participant will receive a numerical identification code, all researchers will sign a data confidentiality commitment sheet prior to the start of the study, which states that the patients’ personal data will be protected.

Each participant’s medical information and progress at each point of measurement shall be recorded in his or her medical record.

## Results

The results will first be expressed by considering ICU stay as the primary variable. In addition, to address secondary variables with criteria, values related to postoperative complications, hospital LOS and quality of life will be cited. This will be followed by values related to functionality and muscular and aerobic endurance. Finally, it will be reflected whether confounding variables may interfere with the direction of the dependent variables.

Quantitative variables will be expressed in terms of mean and standard deviation, provided they comply with a parametric distribution, or in median and interquartile range if this is not the case. Box and whisker plots shall be used for their graphical representation.

Categorical variables, corresponding to the EuroQoL-5D items, will be expressed in terms of frequency and percentages, bar charts will be used for ordinal variables, and nominal descriptive variables will be expressed using pie charts.

## Discussion

In order to provide a better understanding of the most appropriate treatment for patients undergoing heart valve surgery, this will be the first controlled clinical study focused on adding strength exercise as an additional treatment during prehabilitation. This variable will be compared to a protocol focusing on strengthening the respiratory muscles and improving ventilatory capacities, which have already been shown to be effective in patients undergoing major cardiac surgery [9,12,15–17]. To better understand the results, they will be analysed in detail, taking into account the special characteristics of the study sample. They will also be compared with the results reflected in other studies focusing on individuals with the same characteristics and similar objectives, such as LOS or ICU length of stay [25,27], albeit with other types of approaches. Therefore, future studies should analyse our results and transfer the protocol to larger or different population groups.

The results of this study will focus on helping to improve rehabilitation and prehabilitation protocols, considering that it is essential to maintain pulmonary training, as well as the inclusion of peripheral exercises that help people with heart disease to be in a better physical condition in order to increase their participation and sense of quality of life.

If no adverse events are observed in the studied population, future research could consider people with cardiovascular diseases in unstable situations, such as people with valvulopathies attached to non-preserved ventricular ejection. Given the added instability of these people, it would be interesting to consider a more supervised rehabilitation, in terms of hospital interventions or, at least, videoconference supervised rehabilitations if it is not possible for them to travel to the hospital.

## Supporting information

S1 Checklist SPIRIT 2013 checklist: Recommended items to address in a clinical trial protocol and related documents*. (DOC)

S1 Fig Timeline of enrolment, interventions, and assessments. SPIRIT chronogram. Abbreviatures of timepoints: -t1: Enrolment, t0: Alocation, t1: First week before surgery, t2: Second week before surgery, t3: Third week before surgery, t4: 24h after surgery, t5: At hospital discharge, t6: One month after surgery, t7: Six months after surgery.
(TIF)

S2 Fig Flowchart of the patient intervention and measurements. (TIF)

S3 Fig Explanatory diagram of intervention and measurements. (TIF)

S1 File (PDF)
