# Supplementary figures and images for: Can resistance prehabilitation training bring additional benefits in valvular cardiac surgery? protocol for a randomized controlled trial
Source: PLoS One. 2024 May 7;19(5):e0303163. doi: 10.1371/journal.pone.0303163 (PMC11075887; doi:10.1371/journal.pone.0303163)

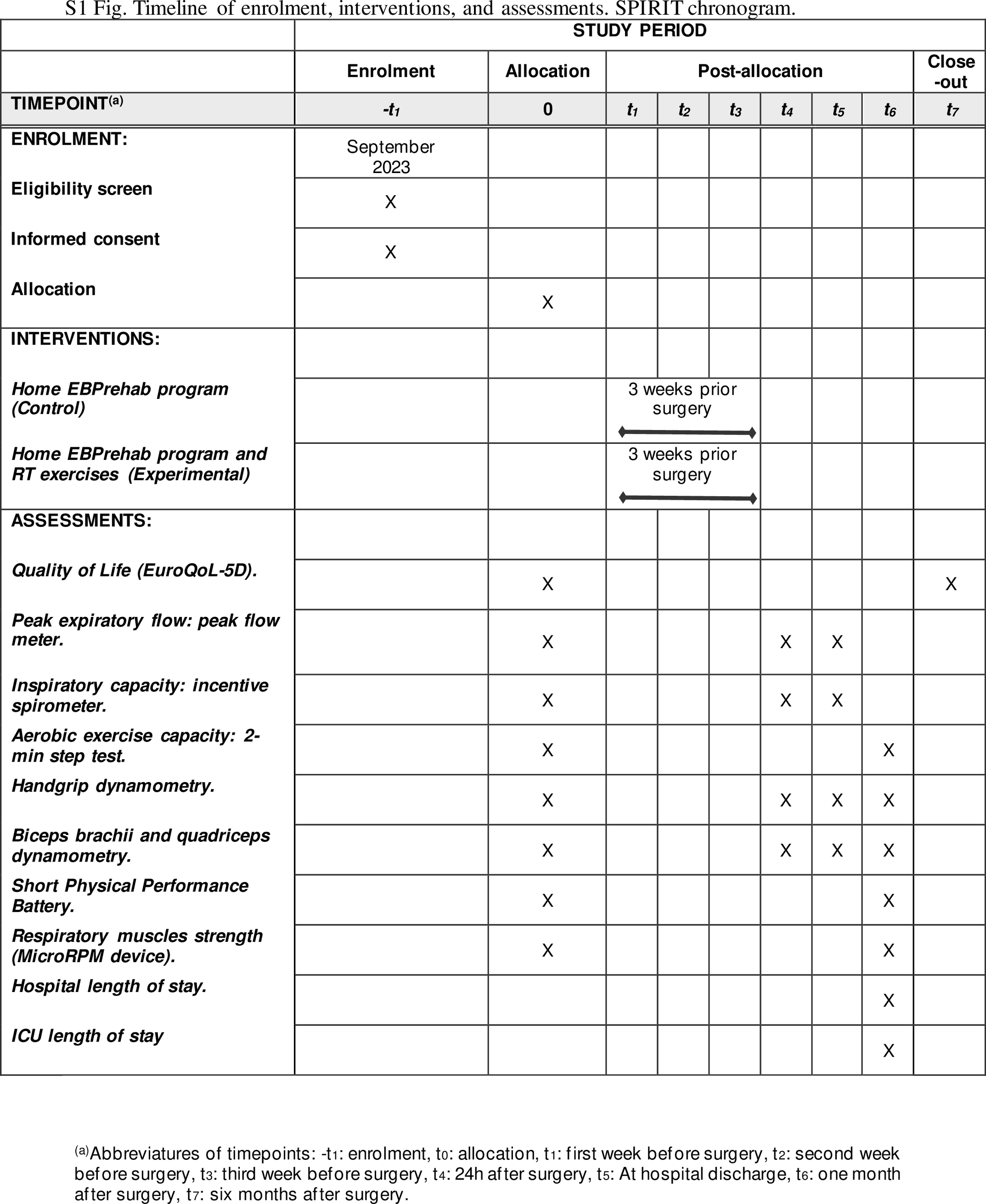

Supplement: S1 Fig — Abbreviatures of timepoints: -t1: Enrolment, t0: Alocation, t1: First week before surgery, t2: Second week before surgery, t3: Third week before surgery, t4: 24h after surgery, t5: At hospital discharge, t6: One month after surgery, t7: Six months after surgery. (TIF) [file pone.0303163.s002.tif]

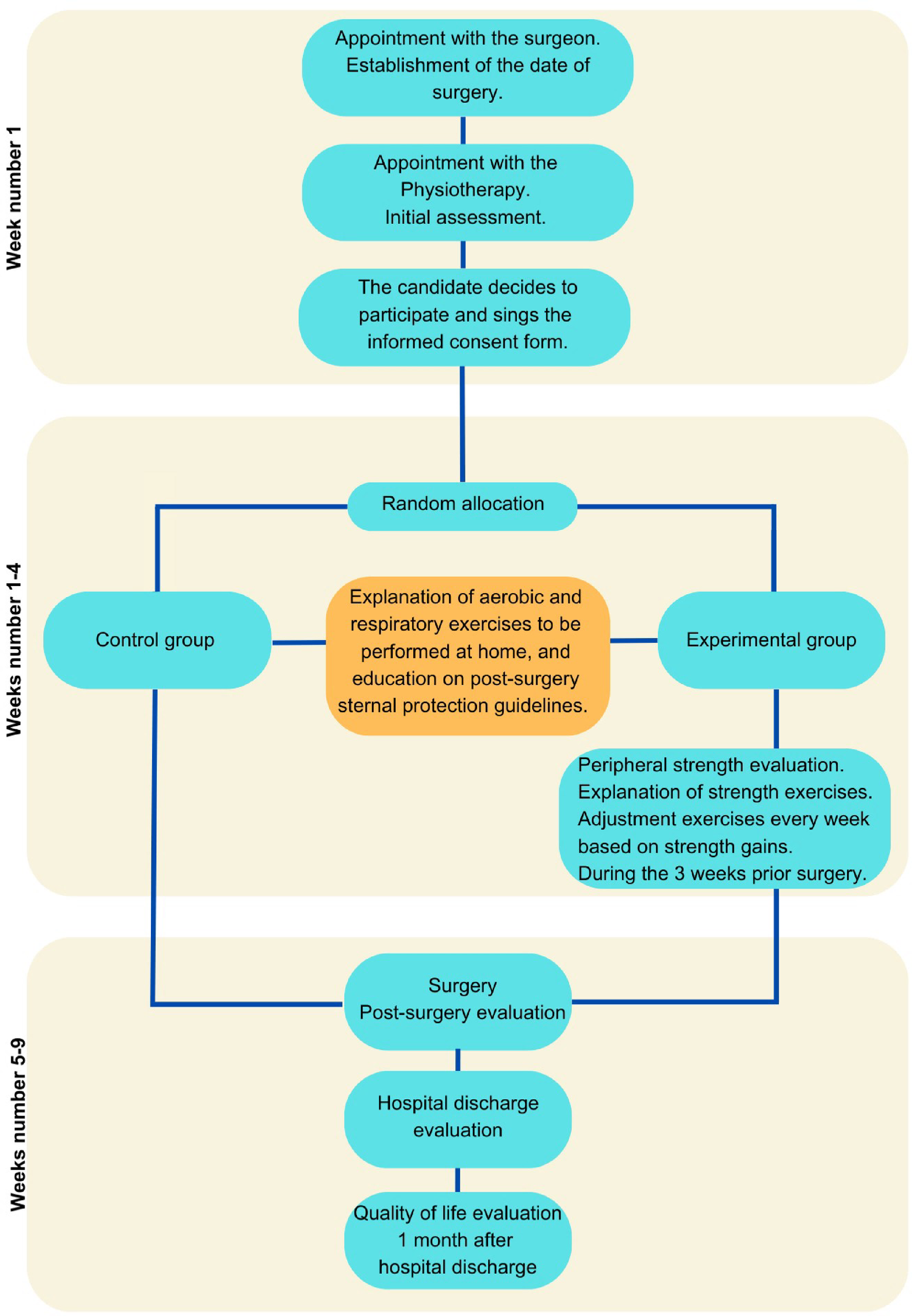

Supplement: S2 Fig — (TIF) [file pone.0303163.s003.tif]

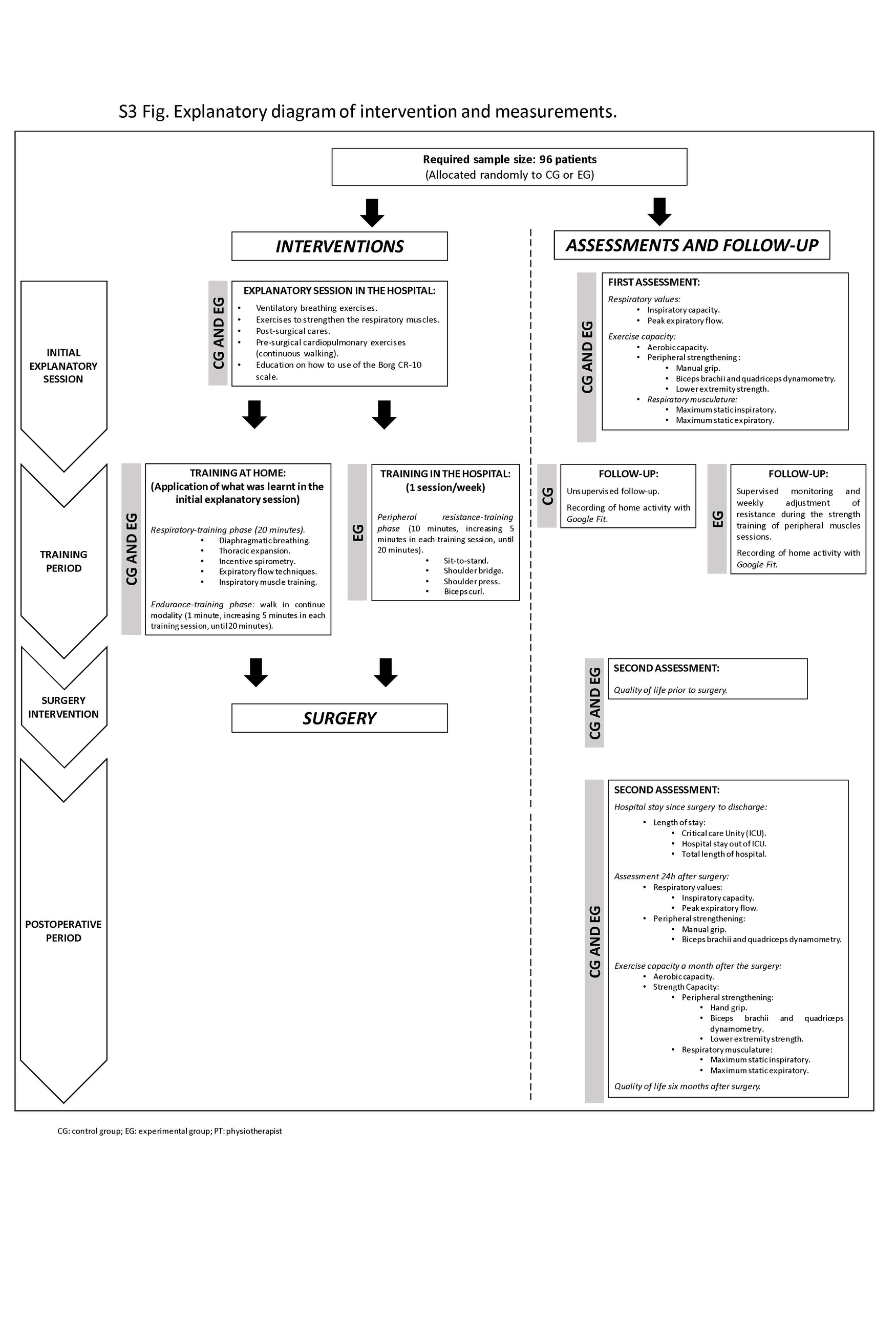

Supplement: S3 Fig — (TIF) [file pone.0303163.s004.tif]
