# Supplementary material for: Can resistance prehabilitation training bring additional benefits in valvular cardiac surgery? protocol for a randomized controlled trial
Source: PLoS One. 2024 May 7;19(5):e0303163. doi: 10.1371/journal.pone.0303163 (PMC11075887; doi:10.1371/journal.pone.0303163)
Supplement: S1 File — (PDF) [file pone.0303163.s005.pdf]

Can resistance training bring additional benefits to a Cardiac Surgery Exercises based Prehabilitation Program? A protocol for a randomized controlled clinical study for valvular heart disease patients. Cardiac EBPrehab.

Jorge Montero Cámara[1] , Francisco José Ferrer Sargues[1*] , María José Segrera Rovira[1,2] , Adrián Sarria Cabello[2] , David Cuesta Pered[o2] , Juan Antonio Margarit Calabuig[2] , María Luz Sánchez Sánchez[3] .

[1]Deparment of Physiotherapy, Universidad Cardenal Herrera-CEU, CEU Universities, Alfara del Patriarca, 46113, Valencia, Spain.

[2]Hospital Universitario de la Ribera, km 1, Ctra. Corbera, 46600 Alzira, Valencia, Spain.

[3]Physiotherapy in Motion. Multispeciality Research Group (PTinMOTION), Department of Physiotherapy, University of Valencia, C/Gascó Oliag n 5, 46010 Valencia, Spain.

*Corresponding author.

INTRODUCTION

Cardiovascular diseases (CVD) include coronary heart disease, cerebrovascular disease, congenital heart disease, deep vein thrombosis, pulmonary embolism, and disorders affecting the heart and blood vessels. (1). A number of risk factors are often associated with acute CVD (1,2) and, at the socio-demographic level, poorer economic or educational conditions may contribute to increased risk factors (3). CVD accounts for 31% of deaths and is the leading cause of mortality worldwide (1,4). (1,4). Coronary heart disease alone accounts for 19% of global deaths in men and 20% in women (1). (1). In Spain, according to the Instituto Nacional de Estadística, CVD caused 24% of all deaths in 2020 (5).

Major surgery is often chosen as the treatment of choice for CVD, with patients often classified as "high risk" due to comorbidities such as frailty, obesity or advanced age. (6). These patient characteristics may increase the likelihood of postoperative pulmonary complications such as pneumothorax, atelectasis, pneumonia, need for prolonged mechanical ventilation, pulmonary oedema or respiratory distress syndrome (7,8).

The concept of *fast-track* rehabilitation after surgery (ERAS, Enhanced recovery after surgery) appeared in the 1970s, and combines different measures to optimise the patient's condition before, during and after surgery in order to allow them to recover their maximum functional capacities as quickly as possible after the surgical procedure. (9-12). Although this concept was initially introduced by surgeons, it is now a transdisciplinary approach that can only be successful with the synergistic involvement of the entire healthcare team. The ERAS (Enhanced Recovery After Surgery) protocol represents a model of patient-centred healthcare collaboration (9,11,13). Despite the name of the protocol, rehabilitation places great emphasis on both preoperative preparation and postoperative re-education (9-13). Furthermore, at a time when controlling health care costs is an ever-present concern, participation in these exercise-based prehabilitation programmes (EBPrehab) may decrease postoperative complications and length of hospital stay (LOS) (9,11,12). (9) improving patients' functional capacity and decreasing healthcare costs (14).

The most commonly used exercises in the pre-surgical intervention of the cardiovascular patient include aerobic training (10,12,15)chest expansion exercises, ventilatory work with a volumetric spirometer or a peak flow meter (9). (9)and exercises to strengthen the respiratory muscles, both inspiratory and expiratory (16,17). The combined work of the exercises described above can reduce complications and LOS (12). (12).

However, beyond ventilatory problems, one of the complications that cardiovascular patients experience after surgery and after their stay in the Intensive Care Unit (ICU) is acquired weakness syndrome, which has a major impact on the peripheral musculature (18). (18). Several studies have linked lower preoperative muscle density to higher postoperative mortality rates (19-21)as well as higher postoperative pulmonary complications (20,22-24). (20,22-24). Similarly, some studies correlate a reduction in manual grip strength with higher mortality, longer LOS, and higher postoperative pulmonary complications (20,22-24). (21) and higher postoperative complications (23,24).

An exercise programme that includes peripheral muscle strengthening, also known as resistance training (RT), has been shown to be effective in reducing in-hospital mortality and LOS in major abdominal surgery. (25,26). RT added to aerobic training can improve quality of life and left ventricular function in people with coronary heart disease (27). However, despite the wide variety of characteristics of cardiac prehabilitation programmes, to our knowledge, none of those published have included RT to study whether, as in other pathologies, there is a therapeutic advantage and a better evolution of patients operated on for valve disease, which would imply the development of a more efficient prehabilitation protocol.

Based on the above, the purpose of the present study is to evaluate whether the implementation of an additional RT prehabilitation protocol within a cardiac EBPrehab (based on aerobic training, ventilatory work and respiratory muscle strengthening) can reduce ICU length of stay, postoperative complications and LOS in valvular patients undergoing cardiac surgery.

In addition, the secondary objective is to find out whether a programme that includes RT in addition to respiratory and aerobic training can have better effects on ventilatory variables compared to a traditional programme based on aerobic exercise, ventilatory work and respiratory muscle strengthening.

METHODOLOGY

Design

A prospective, parallel, non-randomised, single-centre, prospective clinical trial protocol has been designed. In this study, participants will be randomly assigned to either the experimental prehabilitation group or the conventional prehabilitation group during the three weeks prior to surgery.

This study complies with the ethical principles established in the Declaration of Helsinki and is made available to the Ethics Committee of the Hospital de La Ribera. If approved, it will be registered in the U.S. National Library of Medicine (ClinicalTrials.gov).

All candidates will be given and explained an information sheet (Annex I) with detailed information on the study and will be asked for their express consent and signature, after reading and explanation, as a requirement for participation in the study.

Participants: eligibility and recruitment.

Ninety-six patients will participate in this study, whose recruitment is expected to take place between June 2023 and July 2025. The inclusion criteria for the sample will be: adult patients diagnosed with valvular pathology who have been admitted as candidates for cardiac surgery for the first time, and whose appointment for intervention has been scheduled. Only those who voluntarily agree to participate in the study and sign the informed consent form will be admitted. Patients with the following characteristics will be excluded: stage 4 or 5 renal failure, low ejection fraction, Euroscore greater than 15, non-ST-segment elevation acute coronary syndrome (NSTEACS) and coronary artery disease or need for urgent intervention.

Those with cognitive deficit, diagnosis of pulmonary disease, heart failure, unstable angina, arrhythmias, severe aortic stenosis, mitral regurgitation, aortic aneurysm, recent embolism, recent myocarditis or pericarditis, or comorbidities limiting the performance of the functional test will also be excluded.

All persons referred to the Physiotherapy Unit from the Cardiac Surgery Unit of the Hospital de La Ribera (Alzira, Spain) will be considered candidates for this study, as long as they attend the initial physiotherapy assessment. Recruitment will therefore be carried out among these attendees and eligibility will be confirmed by a physiotherapist. Signed informed consent will be collected prior to any measurement or intervention.

The assignment of each participant to one group or another is not the responsibility of the researchers, but will be randomised. The initial and final evaluation will be carried out by three physiotherapists who will act without knowing the assignment of each participant to one or the other group. The physiotherapists who carry out the measurements will attend a training session to unify the measurement criteria for each variable.

Intervention

Figure 1 shows how the intervention and measurement protocol will be developed in each of the groups. Both groups will receive a first session in which they will be instructed to carry out the unsupervised home EBPrehab programme, consisting of respiratory training, ventilatory work and strengthening of the respiratory muscles, aerobic endurance exercise through continuous walking, and a series of measures on post-surgical care. Once the guidelines have been explained, they will be given a series of links to explanatory videos so that they can carry out the work at home seven times a week without supervision. For this intensity adjustment, individual perception of exertion will be used, using a Borg CR-10 scale (29). Participants will be instructed to maintain a moderate intensity, between four and seven on this scale. Aerobic endurance exercises shall be performed in a continuous and harmonious rhythm.

Figure 1. Flow chart. .

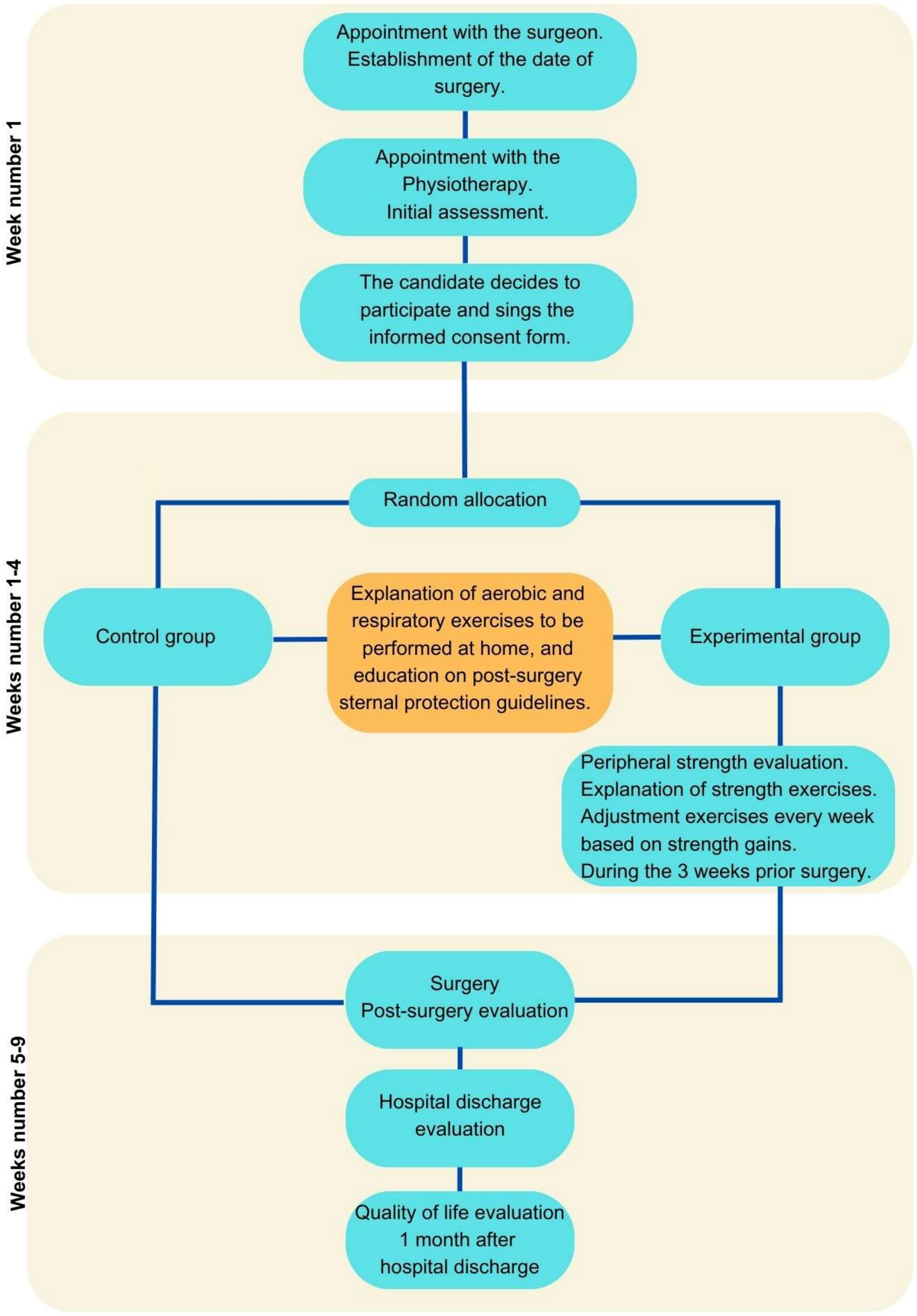

Week number 1
Appointment with the surgeon. Establishment of the date of surgery.
Appointment with the Physiotherapy. Initial assessment.
The candidate decides to participate and sings the informed consent form.
Weeks number 1-4
Random allocation
Control group
Explanation of aerobic and respiratory exercises to be performed at home, and education on post-surgery sternal protection guidelines.
Experimental group
Peripheral strength evaluation.
Explanation of strength exercises.
Adjustment exercises every week based on strength gains.
During the 3 weeks prior surgery.
Weeks number 5-9
Surgery
Post-surgery evaluation
Hospital discharge evaluation
Quality of life evaluation 1 month after hospital discharge

In addition, a preoperative musculoskeletal and cardiopulmonary rehabilitation programme will be implemented in the experimental group for the cardiovascular patient who underwent surgery for valvular heart disease. The intervention has been designed following the guidelines of the American College of Sports Medicine (ACSM) for exercise prescription, considering the FITT-VP principles (Frequency, Intensity, Type, Time, Volume, Progression) for patients with cardiorespiratory pathology and adjusting them to the surgical population. (28). This peripheral muscle strengthening programme (Figure 2) will be modified and adapted in each classroom session, which will require a weekly evaluation and modifications in terms of the load and the number of series and/or repetitions, depending on each participant (29). For this work, participants will also be educated to adjust the intensity using the Borg scale CR-10 (29)by asking them to maintain a moderate intensity, between four and seven on this scale.

Figure 2. Common and group-specific interventions....

Common interventions for both groups

A single explanatory session of:

- Ventilatory breathing exercises.
  - Diaphragmatic breathing.
  - Thoracic expansion exercises.
  - Incentive spirometer.
  - Expiratory flow technique.
- Exercises to strengthen the respiratory muscles.
- Post-surgical care.
- Pre-surgical cardiopulmonary rehabilitation exercises (continuous walking).
- Use of the Borg CR-10 scale.

Control group

- Application at home of what was learnt in the initial explanatory session.
- Unsupervised follow-up.
- Registration of the activity with Google Fit.

Intervention group

- Strength training of peripheral muscles.
- Application at home of what was learnt in the initial explanatory session.
- Supervised monitoring and weekly adjustment of resistance.
- Recording of home activity with Google Fit.

The pre-rehabilitation will be structured in three different phases (Supplementary Material 1):

1. Breathing training phase (Conventional treatment group and experimental group, 20 min). The breathing intervention will include diaphragmatic breathing (3 sets of 10 repetitions), chest expansion exercises (3 sets of 10 repetitions) and incentive spirometry with the Portex® Coach 2® Incentive Spirometer 4000 ml (1 set, with 10 repetitions up to 25% of maximum capacity, and only 1 repetition at maximum inspiratory capacity). Expiratory flow techniques will be taught, using the Sibelmed® Datospir Peak-10 incentive device, and finally, specific training of the respiratory muscles will be carried out using the Threshold® Inspiratory Muscle Trainer

(Respironics, NJ 07054, USA), adjusting the workload to a minimum of 30% of the subject's PIM. (16,30). The workload range of the IMT device will be 9 to 41 cmH2O. The protocol will contain 20 min of training divided into seven sets, with two minutes of work and one minute of rest between sets. Each participant will receive a list of links to demonstration videos and instructions on how to perform each of the exercises.

2. Aerobic endurance training phase (Conventional treatment group and experimental group), 10 min, increasing by five minutes at each training session, up to 20 min. Each patient will walk in a continuous harmonic mode, following the minimum recommendations of the World Health Organisation. (31). Perception of exertion will be assessed using a modified Borg scale.
3. Peripheral resistance training phase (for the experimental group only). 10 min, increasing by five minutes at each training session, up to 20 min. In each session, participants will complete three sets of four exercises. During the first sessions, training will consist of 8 repetitions of the exercises, performed with light resistance bands, and emphasising analytical training of the main muscles (deltoids, biceps brachii, triceps brachii, abdominals, trunk extensors, quadriceps, hamstrings and calves). (32). After the second training session in the hospital, progress to using medium resistance bands and/or increasing repetitions for each exercise. In the final phase, the volume will be increased to 12 repetitions. Each exercise will be rested for 60 seconds.

   To verify that participants perform the exercises at home, regardless of the group to which they are assigned, they will be asked to record their activities at home using the Google Fit application. (33).

The prehabilitation programme common to all participants is the same programme that is conventionally carried out in the prehabilitation service of the Hospital La Ribera. In that sense, although there is no evidence that the exercises proposed before surgery can cause harm to cardiac patients awaiting surgery, to minimise the risks the first session will be carried out in the hospital, adapting the intensity to each individual. Any adverse situation perceived by the patient during the home sessions will be reported. All adverse effects will be evaluated, recorded and discussed in the final article.

Variables

The variables will be divided into four different areas, differentiating between hospital stay, quality of life, respiratory values and exercise capacity (Figure 3).

Figure 1. Variables, tools and timing.

	Hospital stay.	Quality of life.	Respiratory values.	Exercise capacity.
**Outcomes**	Hospital stay since surgery to discharge:<br>• Critical care Unity (ICU).<br>• Hospital stay out of ICU.<br>• Total hospital stay.	Quality of life prior to surgery and six months after surgery	• Peak expiratory flow.<br>• Inspiratory capacity.<br>• Maximum static inspiratory (MIP).<br>• Maximum static expiratory (MEP).	• Aerobic resistance<br>• Lower limb strength.<br>• Hand grip stregth<br>• Biceps brachii and cuadriceps stregth capacity
**Tool**	Accounting of the days according to the data reflected in the medical history.	EuroQoL-5D questionnaire	• Sibelmed Datospir Peak-10.<br>• Portex Coach 2 Incentive Spirometer 4000ml.<br>• MicroRPM device (Carefusion)	• 2 minute step test<br>• Short Physical Performance Battery.<br>• Activity logging via Google Fit<br>• Hand dynamometry.<br>• Dinamometer Lafayette
**Time**	At hospital discharge	Pre-surgery and 6 months after discharge.	Pre-surgery, 24h after the surgery, at the hospital discharge	Pre-surgery, 24h after the surgery, at the hospital discharge

1. <u>Hospital stay.</u> Hospital stay will be measured in hours and minutes, so that stays of less than 24 hours can be better accounted for. Three different variables will be recorded:
    - Intensive Care Unit stay: counted from the end of the surgical procedure until transfer to the ward.
    - Ward stay: counted from transfer to ward until discharge from hospital.
    - Hospital stay: counted from the end of the surgical procedure until discharge from hospital.
2. <u>Quality of life.</u> Perception of quality of life will be recorded and assessed both before and six months after surgery. The EuroQOL-5D quality of life questionnaire, valid and reliable for analysing quality of life in this type of patient, will be used. The participant assesses their capacity for self-care, mobility, activities of daily living, the presence of pain or anxiety/depression in categorical levels of severity (no problems, some problems or moderate problems and severe problems) and their perception of health using a visual analogue scale. (34). As there are no published data on the inter-examiner reliability of this tool (35)a single nurse will be responsible for collecting these values.
3. <u>Respiratory values.</u> Respiratory values shall be collected on three different occasions: before surgery, immediately after surgery and on the day of hospital discharge. These tests shall be repeated until three acceptable measurements are obtained (difference <10%), with a one-minute rest between them, and the highest value shall be recorded (36,37). Three measurements shall be made of:

- Inspiratory capacity: This is defined as the volume of air that can be inspired from the resting inspiratory position. With the patient in a seated position, the Portex® Coach 2® 4000 ml incentive spirometer will be used to measure inspiratory capacity and the value of the variable will be expressed in millilitres. The patient will be instructed to take a deep, unforced inspiration, trying not to exceed the flow mark (38).
- Peak expiratory flow: This is defined as the maximum flow a person is able to exhale during a brief maximal expiratory effort after a full inspiration. With the patient in a seated position, the Sibelmed® Datospir Peak-10 device will be used to record the peak expiratory flow and the value of the variable will be expressed in litres/second. The patient will be instructed in a maximal forced expiration, preceded by a deep nasal inspiration up to inspiratory reserve volume (39).
- Respiratory pressures: These are used to calculate the strength of the respiratory muscles, both at the diaphragmatic (inspiratory) and abdominal and intercostal (expiratory) levels. Maximum static inspiratory pressures (MIP) and maximum static expiratory pressures (MEP) will be measured in a seated position, using a MicroRPM device (Carefusion, VYAIRE MEDICAL, UK). A careful explanation of the test will be given, encouraging the patient to perform a maximal inspiration and inspiration of approximately 2-3 seconds against the resisted device. Predicted values will be estimated using the equation proposed by Heinzmann et al.40).

4. Exercise capacity. Exercise capacity and endurance will be collected before, 24 hours after surgery and at discharge. The tests used will be:
    - Aerobic capacity: will be assessed by the 2-minutes Step Test, a valid tool to assess functionality in patients with cardiac pathology after surgery and sensitive to assess perioperative changes. (41)which also has excellent inter-examiner reliability (41). (41). Following the protocol established by Jones and Rikli (42)implemented in 2022 by Chow et al. in revascularised individuals (41)the aim of this test is to count the number of repetitions that each participant is able to perform in two minutes. In case a person needs technical assistance to perform the test, a stable chair will be placed close to him/her to limit the risk of falling. The assessor shall use a manual counter to record the number of steps each person takes by lifting one knee to the middle distance between the patella and the anterior superior iliac spine. (42).
    - Manual grip strength: grip strength capacity shall be recorded with manual dynamometry (kg), using a Jamar Plus+® device, which is reliable for measuring changes in patients with cardiac disease, and whose minimum detectable change is 5.2 kg and 5.1 kg for the right and left hand, respectively. (43). With the patient in a seated position, a maximum hand closure is requested for 2-3 seconds, the hand is changed, and the operation is repeated up to 3 times, with a difference of less than 10% between measurements. This tool has inter-examiner reliability (44).

- Analytical strength of the biceps brachii and quadriceps femoris: The strength of the biceps brachii and quadriceps femoris will be measured on the arms and legs with a Dynamometer Lafayette Manual Muscle Tester (Lafayette, IN, USA). (45,46). The measurement technique was described by Bohannon, who verified the excellent intra- and inter-examiner reliability of this tool (47).
- Lower extremity strength: lower extremity strength will be assessed using the Short Physical Performance Battery, a functional test battery that is simple, valid, reliable, sensitive to clinical functional changes and with adequate intra- and inter-examiner reliability (48,49). (48,49). The test consists of three parts:
    - A five-squat test in a standing position, reliable for measuring change, with a minimum detectable change of 3.12 seconds. (43). The time taken to perform the five squats will be categorised according to the following criteria: time less than or equal to 11.19 seconds; between 11.20 and 13.69 seconds; between 13.70 and 16.69 seconds; between 16.70 and 60 seconds; greater than 60 seconds or inability to perform.
    - Static balance, where the participant is asked to adopt three positions during standing: feet together, semi-tandem and tandem. It is quantified if the time they can hold the position is less than or at least 10 seconds.
    - Gait ability, where the patient will be asked to walk in a 4m corridor at a normal pace and the best time in seconds of two attempts will be recorded. Gait ability will be categorised as follows: Unable to perform, time greater than 8.7 seconds; time less than 8.7 seconds, but equal to or greater than 6.21 seconds; time less than 6.20 seconds, but greater than or equal to 4.82 seconds; time less than 4.82 seconds.

<u>Statistical analysis.</u>

An independent statistician will carry out the statistical analysis. He or she will have no knowledge of whether each participant belongs to one group or another, nor what type of treatment was implemented in each group. All necessary data will be extracted into an Excel spreadsheet and analysed using IBM® SPSS® Statistics 27.0.1.0.

A Kolmogorov-Smirnov test will be used to check whether or not the data fit a normal distribution. A descriptive analysis will be carried out to explore the variables related to the study.

Quantitative variables with parametric distribution will be assessed with a T-test or ANOVA, while if the distribution is non-parametric a Mann-Whitney test will be used. This analysis will examine whether there are differences between groups that may support or refute the hypothesis that strength training reduces hospital stay, ICU stay and/or complications in this type of patient. This analysis will also be used to test whether ventilation and respiration values as well as quality of life values are different in each group.

To assess whether there is an influence of confounding variables (BMI, toxic habits, comorbidities, underlying pathologies) on the direction of the dependent variables, multiple regression will be performed.

Sample size.

For the primary outcome (time in the intensive care unit), an effect size of 0.613 was determined based on previous research on the effect of a multidimensional preoperative intervention in patients awaiting elective coronary artery bypass graft surgery (47). To obtain a statistical power of 0.8 with a 2-tailed alpha of 0.05 in a Student's t-test, considering a dropout rate of 10%, a total of 96 participants (48 subjects per group) were required.

Data collection

Sex, age, underlying pathology, comorbidities, body mass index (BMI) and toxic habits will be the descriptive variables collected in both groups. All these data will be stored in their clinical history and will be extracted by the person in charge of epidemiological data management at the hospital, who will include them in an Excel table, also indicating whether each participant survives or not, as well as the information related to the intervention and the pathology, catalogued using the International Classification of Diseases 11 (ICD-11) or the appropriate one if there are updates. Days of hospitalisation, intensive care unit (ICU) stay, if applicable, Charlson comorbidity index, and possible postoperative complications for each participant will also be reflected in this table.

Although the database from which the data will be extracted will not contain personal data, as each participant will receive a numerical identification code, all researchers will sign a data confidentiality commitment sheet prior to the start of the study, which states that patients' personal data will be protected.

The medical information of each participant and his or her evolution at each point of the measurement shall be recorded in his or her medical record.

RESULTS

The results will first be expressed taking into account hospital and/or ICU stay as the primary variable. Also, to address secondary variables with criteria, values related to postoperative complications and quality of life will be cited. This will be followed by values related to functionality and muscular and aerobic endurance. Finally, it will be reflected whether confounding variables may interfere with the direction of dependent variables.

Quantitative variables will be expressed in terms of means and standard deviations, provided that they comply with a parametric distribution, or in medians and interquartile ranges if this is not the case. Box and whisker plots shall be used for their graphical representation.

Categorical variables, corresponding to the EuroQoL-5D items, will be expressed in terms of frequency and percentages and bar charts will be used for ordinal variables, while nominal descriptive variables will be expressed using pie charts.

DISCUSSION

In order to provide a better understanding of the most appropriate treatment for patients undergoing heart valve surgery, this will be the first controlled clinical study focused on adding strength exercise as an additional treatment during prehabilitation. This variable will be compared to a protocol focusing on strengthening respiratory muscles and improving ventilatory capabilities, which have already been shown to be effective in people undergoing major cardiac surgery (9,12,15-17). To better understand the results, they will be carefully analysed, taking into account the special characteristics of the study sample. They will also be compared with the results reflected in other studies focusing on individuals with the same characteristics and focusing on similar objectives, albeit with other types of approaches. Therefore, future studies will be able to analyse our results and transfer the protocol to larger or different population groups.

The results of this study focus on helping to improve rehabilitation and prehabilitation protocols, considering that it is important to maintain pulmonary training, as well as the inclusion of peripheral exercises that help people with heart disease to be in better physical condition in order to increase their participation and sense of quality of life.

Supplementary Material 1. Intervention in Physiotherapy.

**Training common to both groups**					**Specific training of the experimental group**			
**Respiratory-training phase**				**Endurance-training phase**	**Peripheral resistance-training phase**			
20 minutes				10 minutes, increasing 5 minutes in each training session, until 20 minutes.	10 minutes, increasing 5 minutes in each training session, until 20 minutes.			
Exercise	Series	Reps.	Rest	Exercise	Exercise	Series	Reps.	Rest
Diapragmatic breathing	3	10	1 min.	Walk in continue modality. Perceived exertion will be important, so we will give a modified-Borg scale to all patients.	Sit-to-stand	3	8-12	20 sec
Thoracic expansion	3	10	1 min.		Shoulder bridge	3	8-12	20 sec
incentive spirometry	1	10<br>25% LC	1 min.		Shoulder press	3	8-12	20 sec
incentive spirometry	1	1<br>100% MC	1 min.		Biceps curl	3	8-12	20 sec
Expiratory flow techniques			1 min.					
Inspiratory muscle training	1	30% MIP	1 min.					

BIBLIOGRAPHY

1. Lennon RP, Claussen KA, Kuersteiner KA. State of the Heart: An Overview of the Disease Burden of Cardiovascular Disease from an Epidemiologic Perspective. Prim Care 2018;45:1-15.
2. Reamy B v., Williams PM, Kuckel DP. Prevention of Cardiovascular Disease. Prim Care 2018;45:25-44.
3. Rodriguez-Alvarez E, Lanborena N, Borrell LN. Cardiovascular disease risk factors in Spain: A comparison of native and immigrant populations. PLoS One 2020;15:e0242740.
4. GBDCoD Collaborators. Global, regional, and national age-sex-specific mortality for 282 causes of death in 195 countries and territories, 1980-2017: a systematic analysis for the Global Burden of Disease Study 2017 GBD 2017 Causes of Death Collaborators*. Lancet 2018;392:1736-88.
5. INE. National Statistics Institute. Deaths by cause of death. Year 2020. INE Press Releases 2021.
6. Kamarajah SK, Bundred J, Weblin J, Tan BHL. Critical appraisal on the impact of preoperative rehabilitation and outcomes after major abdominal and cardiothoracic surgery: A systematic review and meta-analysis. Surgery (United States) 2020;167:540-9.
7. Cavayas YA, Eljaiek R, Rodrigue É, Lamarche Y, Girard M, Wang HT, et al. Preoperative Diaphragm Function Is Associated with Postoperative Pulmonary Complications after Cardiac Surgery. Crit Care Med 2019;47:e966-74.
8. Miskovic A, Lumb AB. Postoperative pulmonary complications. Br J Anaesth 2017;118:317-34.
9. Dinic VD, Stojanovic MD, Markovic D, Cvetanovic V, Vukovic AZ, Jankovic RJ. Enhanced recovery in thoracic surgery: A review. Front Med 2018;5.
10. Steinmetz C, Bjarnason-Wehrens B, Baumgarten H, Walther T, Mengden T, Walther C. Prehabilitation in patients awaiting elective coronary artery bypass graft surgery - effects on functional capacity and quality of life: a randomized controlled trial. Clin Rehabil 2020;34:1256-67.
11. Ljungqvist O, Scott M, Fearon KC. Enhanced recovery after surgery a review. JAMA Surg 2017;152:292-8.
12. Steinmetz C, Bjarnason-Wehrens B, Walther T, Schaffland TF, Walther C. Efficacy of Prehabilitation Before Cardiac Surgery. Am J Phys Med Rehabil 2023;102:323-30.
13. Brunelli A. Enhanced recovery after surgery in thoracic surgery: the past, the present and the future. Video Assist Thorac Surg 2017;2:37-37.
14. Billard V, Estebe J-P, Ecoffey C. Rapid rehabilitation after surgery. Anesthesia & Resuscitation 2015;1:397-400.
15. Barakat HM, Shahin Y, Barnes R, Gohil R, Souroullas P, Khan J, et al. Supervised exercise program improves aerobic fitness in patients awaiting abdominal aortic aneurysm repair. Ann Vasc Surg 2014;28:74-9.
16. Hulzebos EHJ, Helders PJM, Favié NJ, De Bie RA, Brutel De La Riviere A, Van Meeteren NLU. Preoperative Intensive Inspiratory Muscle Training to Prevent Postoperative Pulmonary Complications in High-Risk Patients Undergoing CABG Surgery A Randomized Clinical Trial. JAMA 2006;296:1851-7.

17. Dsouza FV, Amaravadi SK, Samuel SR, Raghavan H, Ravishankar N. Effectiveness of Inspiratory Muscle Training on Respiratory Muscle Strength in Patients Undergoing Cardiac Surgeries: A Systematic Review With Meta-Analysis. Ann Rehabil Med 2021;45:264-73.

18. Mendelson AA, Erickson D, Villar R. The role of the microcirculation and integrative cardiovascular physiology in the pathogenesis of ICU-acquired weakness. Front Physiol 2023;14.

19. van Erck D, Dolman CD, Limpens J, Scholte op Reimer WJM, Henriques JP, Delewi R, et al. Preprocedural muscle strength and physical performance and the association with functional decline or mortality in frail older patients after transcatheter aortic valve implementation: a systematic review and meta-analysis. Age Ageing 2022;51.

20. Yamashita M, Kamiya K, Matsunaga A, Kitamura T, Hamazaki N, Nozaki K, et al. Low skeletal muscle density combined with muscle dysfunction predicts adverse events after adult cardiovascular surgery. Nutrition, Metabolism and Cardiovascular Diseases 2021;31:1782-90.

21. Panagidi M, Papazoglou AS, Moysidis D V., Vlachopoulou E, Papadakis M, Kouidi E, et al. Prognostic value of combined preoperative phase angle and handgrip strength in cardiac surgery. J Cardiothorac Surg 2022;17.

22. Sugimoto N, Kuhara S, Nawata K, Yano Y, Teramatsu H, Itoh H, et al. Preoperative decline in skeletal muscle strength of patients with cardiovascular disease affects postoperative pulmonary complication occurrence: a single-center retrospective study. Heart Vessels 2022.

23. Li L, Yang Q, Guo Q, Liu D, Gao H, Liu Y. Preoperative physical performance predicts pulmonary complications after coronary artery bypass grafting: a prospective study. Sci Rep 2022;12.

24. Fu L, Zhang Y, Shao B, Liu X, Yuan B, Wang Z, et al. Perioperative poor grip strength recovery is associated with 30-day complication rate after cardiac surgery discharge in middle-aged and older adults - A prospective observational study. BMC Cardiovasc Disord 2019;19.

25. Dronkers JJ, Chorus AMJ, Van Meeteren NLU, Hopman-Rock M. The association of pre-operative physical fitness and physical activity with outcome after scheduled major abdominal surgery. Anaesthesia 2013;68:67-73.

26. Peng LH, Wang WJ, Chen J, Jin JY, Min S, Qin PP. Implementation of the pre-operative rehabilitation recovery protocol and its effect on the quality of recovery after colorectal surgeries. Chin Med J (Engl) 2021;134:2865-73.

27. Fan Y, Yu M, Li J, Zhang H, Liu Q, Zhao L, et al. Efficacy and Safety of Resistance Training for Coronary Heart Disease Rehabilitation: A Systematic Review of Randomized Controlled Trials. Front Cardiovasc Med 2021;8.

28. American College of Sports Medicine. ACSM's Guidelines for Exercise Testing and Prescription. 10th ed. Wolters Kluwer. Available Online: Https://ShopLwwCom/ACSM-s-Guidelines-for-Exercise-Testing-and-Prescription/p/9781496339065 2017.

29. Borg G. Borg's Perceived Exertion And Pain Scales. 1998.

30. Kendall F, Oliveira J, Peleteiro B, Pinho P, Bastos PT. Inspiratory muscle training is effective to reduce postoperative pulmonary complications and length of hospital stay: a systematic review and meta-analysis. Disabil Rehabil 2018;40:864-82.

31. Bull FC, Al-Ansari SS, Biddle S, Borodulin K, Buman MP, Cardon G, et al. World Health Organization 2020 guidelines on physical activity and sedentary behaviour. Br J Sports Med 2020;54:1451-62.

32. Waite I, Deshpande R, Baghai M, Massey T, Wendler O, Greenwood S. Home-based preoperative rehabilitation (prehab) to improve physical function and reduce hospital length of stay for frail patients undergoing coronary artery bypass graft and valve surgery. J Cardiothorac Surg 2017;12.

33. Scherrenberg M, Bonneux C, Mahmood DY, Hansen D, Dendale P, Coninx K. A Mobile Application to Perform the Six-Minute Walk Test (6MWT) at Home: A Random Walk in the Park Is as Accurate as a Standardized 6MWT. Sensors 2022;22.

34. De Smedt D, Clays E, Doyle F, Kotseva K, Prugger C, Pająk A, et al. Validity and reliability of three commonly used quality of life measures in a large European population of coronary heart disease patients. Int J Cardiol 2013;167:2294-9.

35. Feng YS, Kohlmann T, Janssen MF, Buchholz I. Psychometric properties of the EQ-5D-5L: a systematic review of the literature. Quality of Life Research 2021;30:647-73.

36. American Thoracic Society/European Respiratory Society. ATS/ERS Statement on respiratory muscle testing. Am J Respir Crit Care Med 2002;166:518-624.

37. Laveneziana P, Albuquerque A, Aliverti A, Babb T, Barreiro E, Dres M, et al. ERS statement on respiratory muscle testing at rest and during exercise. Eur Respir J 2019;53:1801214.

38. Graham BL, Steenbruggen I, Barjaktarevic IZ, Cooper BG, Hall GL, Hallstrand TS, et al. Standardization of spirometry 2019 update an official American Thoracic Society and European Respiratory Society technical statement. Am J Respir Crit Care Med 2019;200:E70-88.

39. Self TH, George CM, Wallace JL, Patterson SJ, Finch CK. Incorrect use of peak flow meters: Are you observing your patients? Journal of Asthma 2014;51:566-72.

40. Heinzmann-Filho JP, Vidal PCV, Jones MH, Donadio MVF. Normal values for respiratory muscle strength in healthy preschoolers and school children. Respir Med 2012;106:1639-46.

41. Chow JJL, Fitzgerald C, Rand S. The 2 min step test: A reliable and valid measure of functional capacity in older adults post coronary revascularisation. Physiotherapy Research International 2022.

42. Jones CJ, Rikli RE. Measuring functional. The Journal on Active Aging 2002;1:24-30.

43. Puthoff ML, Saskowski D. Reliability and Responsiveness of Gait Speed, Five Times Sit to Stand, and Hand Grip Strength for Patients in Cardiac Rehabilitation. Cardiopulm Phys Ther J 2013;24:31-7.

44. Härkönen R, Harju R, Alaranta H. Accuracy of the Jamar Dynamometer. Journal of Hand Therapy 1993;6:259-62.

45. Ferrer-Sargues FJ, Peiró-Molina E, Cebrià I Iranzo MÀ, Carrasco Moreno JI, Cano-Sánchez A, Vázquez-Arce MI, et al. Effects of cardiopulmonary rehabilitation on the muscle function of children with congenital heart disease: A prospective cohort study. Int J Environ Res Public Health 2021;18.

46. Stark T, Walker B, Phillips JK, Fejer R, Beck R. Hand-held dynamometry correlation with the gold standard isokinetic dynamometry: A systematic review. PM and R 2011;3:472-9.

47. Bohannon RW. Test-retest reliabilty of hand-held dynamometry during a single session of strength assessment. Phys Ther 1986;66:206-8.

48. Ferronato L, Vieira D, Rodrigues A, Coronel C, de Avelar NP. Short physical performance battery in the pre and postoperative myocardial revascularization surgery in older adults: Reliability, hemodynamic responses, subjective perceived exertion, and adverse events. Physiother Theory Pract 2022.

49. Miyata K, Igarashi T, Tamura S, Iizuka T, Otani T, Usuda S. Rasch analysis of the Short Physical Performance Battery in older inpatients with heart failure. Disabil Rehabil 2023.

50. Heather AM, Daniels C, Mckelvie R, Hirsh J, Rush B. Effect of a Preoperative Intervention on Preoperative and Postoperative Outcomes in Low-Risk Patients Awaiting Elective Coronary Artery Bypass Graft Surgery A Randomized, Controlled Trial. Ann Intern Med 2000;133:253-62.
